# Maximum-likelihood method identifies meiotic restitution mechanism from heterozygosity transmission of centromeric loci: application in citrus

**DOI:** 10.1038/srep09897

**Published:** 2015-04-20

**Authors:** José Cuenca, Pablo Aleza, José Juárez, Andrés García-Lor, Yann Froelicher, Luis Navarro, Patrick Ollitrault

**Affiliations:** 1 Crop Protection and Biotechnology Center. Instituto Valenciano de Investigaciones Agrarias (IVIA); 2 Centre de Coopération Internationale en Recherche Agronomique pour le Développement (CIRAD)

## Abstract

Polyploidisation is a key source of diversification and speciation in plants. Most researchers consider sexual polyploidisation leading to unreduced gamete as its main origin. Unreduced gametes are useful in several crop breeding schemes. Their formation mechanism, i.e., First-Division Restitution (FDR) or Second-Division Restitution (SDR), greatly impacts the gametic and population structures and, therefore, the breeding efficiency. Previous methods to identify the underlying mechanism required the analysis of a large set of markers over large progeny. This work develops a new maximum-likelihood method to identify the unreduced gamete formation mechanism both at the population and individual levels using independent centromeric markers. Knowledge of marker-centromere distances greatly improves the statistical power of the comparison between the SDR and FDR hypotheses. Simulating data demonstrated the importance of selecting markers very close to the centromere to obtain significant conclusions at individual level. This new method was used to identify the meiotic restitution mechanism in nineteen mandarin genotypes used as female parents in triploid citrus breeding. SDR was identified for 85.3% of 543 triploid hybrids and FDR for 0.6%. No significant conclusions were obtained for 14.1% of the hybrids. At population level SDR was the predominant mechanisms for the 19 parental mandarins.

Polyploidisation is a key source of species diversification and speciation in plants^1^,^2^,^3^ and may occur by somatic chromosome doubling (somatic polyploidisation) or sexually through gametic nonreduction (sexual polyploidisation)^4^. Currently, most researchers consider sexual polyploidisation, leading to unreduced gamete, to be the main mechanism of polyploidisation in plants^1^,^5^,^6^.

Meiotic aberrations related to spindle formation, spindle function and cytokinesis can lead to unreduced gamete formation in plants. Up to seven major mechanisms of 2*n* gamete formation have been cytogenetically characterised: premeiotic doubling, first-division restitution (FDR), chromosome replication during the meiotic interphase, second-division restitution (SDR), postmeiotic doubling, indeterminate meiotic restitution, and apospory^7^,^8^,^9^. However, FDR and SDR are the predominant mechanisms of 2*n* gamete formation^4^. Failure of the first (FDR) or second (SDR) divisions leads to the formation of restitution nuclei with an unreduced chromosome number. A FDR 2*n* gamete contains non-sister chromatids, while a SDR 2*n* gamete contains two sister chromatids^5^,^10^,^11^.

The use of unreduced gametes in plant breeding^9^,^12^, resulting in the establishment of sexual polyploids, is useful for improvement of crops such as lily^8^,^13^,^14^, maize^15^, potato^16^,^17^,^18^, rose^19^, rye^20^, alfalfa^21^,^22^, banana^23^,^24^ and citrus^25^,^26^,^27^,^28^,^29^.

Diploidy is the general rule in *Citrus* and its related genera, with a basic chromosome number x = 9^30^. However, triploid breeding has become an important strategic tool in the development of new seedless citrus commercial varieties^25^,^26^,^27^,^28^,^29^. Indeed, seedlessness is one of the most important economic traits related to fruit quality for fresh-fruit marketing of mandarins^26^,^27^,^31^. Very large triploid progenies have been obtained from 2*x* × 2*x* crosses^32^ and several cultivars patented^28^,^29^.

Cytogenetic studies^33^ showed that triploid embryos are associated with pentaploid endosperm, indicating that triploid hybrids result from the fertilisation of unreduced ovules by normal haploid pollen. According to the genotype, the frequency of duplication in the female gametes can range from below 1% to over 20%. Esen *et* *al.*^34^ proposed that, citrus, 2*n* eggs result from the abortion of the second meiotic division in the megaspore. This hypothesis was corroborated by molecular marker analysis for clementine (*Citrus clementina* Hort. ex Tan.)^35^,^36^. The method proposed by Cuenca *et* *al.*^37^ was successfully applied in populations of 2*n* ovules of ‘Fortune’ mandarin and ‘Nules’ clementine, and it was concluded that SDR was the main restitution mechanism and that partial chromosome interference occurs^36^,^37^. By contrast, Chen *et* *al*.^38^ proposed that 2*n* eggs of sweet orange (*C. sinensis* (L.) Osb.) resulted from first meiotic division restitution.

The origin of 2*n* gamete formation greatly impacts the gametic structures and, therefore, the polyploid populations and the efficiency of breeding strategies. Under FDR, non-sister chromatids retain parental heterozygosity from the centromere to the first crossover point,. Under SDR, the two sister chromatids are homozygous between the centromere and the first crossover point (Figure 1^5^). As a consequence, several studies based on genetic markers indicate that FDR gametes transmit 70–80% of the parental heterozygosity, but SDR gametes transmit only 30–40%^9^,^19^,^39^,^40^,^41^,^42^. Thus, a tighter distribution is expected in FDR-derived populations than in SDR ones because a higher percentage of the parental genome is transferred intact, resulting in a more uniform gamete production^43^. Therefore, insights into the meiotic nuclear restitution mechanisms that produce unreduced gametes are crucial for the optimisation of breeding strategies based on sexual polyploidisation^44^.

The identification of the mechanisms driving the formation of 2*n* gametes is complex. However, the use of cytological or marker analysis on polyploid progeny provide accurate or additional information on these mechanisms^9^,^19^,^45^. Molecular cytological approaches have been used successfully, including the unequivocal identification of genomes and recombinant segments in the sexual polyploid progenies^11^,^14^,^45^,^46^,^47^. Molecular marker analysis is also a valuable tool for the estimation of parental heterozygosity restitution (HR) through diploid gametes to polyploid progenies and, therefore, to identify the mechanisms underlying unreduced gamete formation^22^,^35^,^38^,^39^,^41^,^48^,^49^. Several previously developed methods are based on the analysis of HR rates for randomly chosen unmapped markers^38^. These methods require the analysis of a large set of molecular markers to encounter, by chance, the loci with HR lower than 50% that are only found under SDR^50^. However, when HR over 50% is observed for all loci, no definitive conclusion can be reached without a prior knowledge of their location relative to a centromere. Significant FDR conclusions are therefore difficult to obtain with such non-mapped markers. Half-tetrad analysis (HTA;^51^, based on multiple linked loci, is a powerful method for mapping centromeres or for determining the mode(s) of 2*n* gamete formation. Tavoletti *et* *al.*^10^ developed a multilocus maximum-likelihood method of HTA that permits the estimation of both the relative frequencies of FDR and SDR 2*n* gametes and the centromere location within a linkage group without relying on previously identified centromeric markers. The models described therein are all based on population analysis and suppose complete chiasma interference.

Cuenca *et* *al.*^37^ proposed an approach that takes into account different models of chromosome interference (i.e., no interference, partial interference or complete chiasma interference) when testing for FDR and SDR, and for mapping centromeres to linkage groups. This approach is based on functions of heterozygosity restitution (HR) at the population level along a chromosome in relation to locus-centromere distance (d)^52^. Indeed, under FDR or SDR, HR is a direct function of the crossing over frequency between the considered locus and the centromere. It is, therefore, possible to implement the function (HR = f(d)) according to the FDR and SDR hypotheses while also taking into account different models of chromosome interference (Figure 2).

In the present work, we propose a maximum-likelihood approach to test the SDR/FDR mechanism based on the HR of unlinked markers located close to the centromere of different chromosomes. This approach can be applied at the individual or population level. We simulated 2*n* gamete populations arising from FDR or SDR. This enabled us to identify the number of independent markers necessary to test in order to draw significant conclusions at the individual level in relation to marker/centromere distances, as well as the minimum population size necessary to be able to draw significant conclusions when analysing a defined number of unlinked markers.

As a concrete application this new method has been used for investigating the unreduced gamete formation in citrus. Taking advantage of the centromere locations^36^ within the nine linkage groups of the clementine reference genetic map^53^, we selected centromeric markers and used the proposed maximum-likelihood method to (i) check the potential variability of origin between individuals for two genotypes in which SDR was proposed to be the predominant polyploidisation mechanism as determined by population analysis (‘Fortune’ mandarin^37^, and clementine^35^,^36^, and (ii) shed light on the mechanism leading to unreduced gamete formation in a range of mandarin genotypes used as female parents in 2*x* × 2*x* triploid breeding programs.

## Results

### Statistical method for the identification of meiotic restitution mechanism

#### Identification of the restitution mechanism at an individual level

For loci heterozygous for the parent producing the 2*n* gamete, the probabilities of a 2*n* gamete being heterozygous or homozygous as a consequence of FDR or SDR mechanisms are direct functions of the marker-centromere distance.

**Figure 2:**
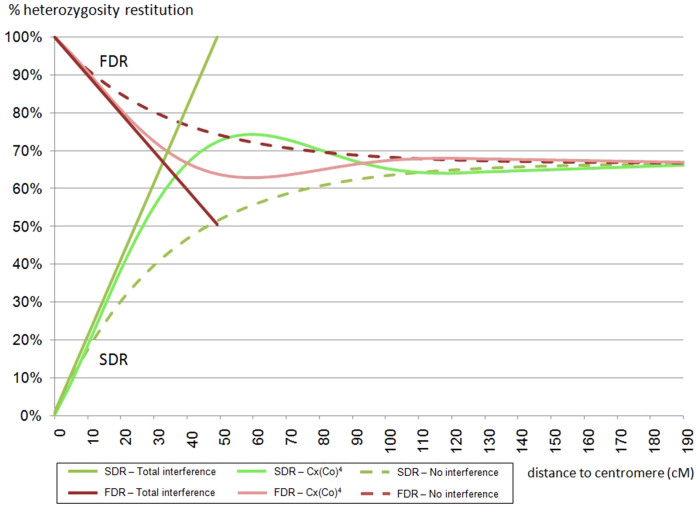
Rate (percentage) of heterozygosity restitution in the unreduced gametes under FDR and SDR mechanisms in function of the locus-centromere distance considering the total interference model, the no interference model and the Cx(Co)^4^ partial interference model (adapted from Cuenca *et* *al*.^37^).

To estimate such probabilities, the function relating HR rate and locus-centromere distance^37^, derived from the *Cx(Co)*^4^ partial chiasma interference model developed by Zhao and Speed^52^ and Foss *et* *al.*^54^, could be used. Indeed, Cuenca *et* *al.*^37^ showed that this model fit better to ‘Fortune’ mandarin data (SDR mechanism) than total or no interference models. However, since selected markers are located close to centromeres (as explained above), for our data, the *Cx(CO)*^4^ model and the total interference model are equivalent (Figure 2). To simplify mathematical calculations of probabilities, the total interference model was used. Marker-centromere distances (d) in Morgan units were estimated from the centromere locations^36^ in the clementine reference genetic map^53^.

The probabilities of a marker being inherited as heterozygous under the SDR [P_SDR_(M_He_)] or FDR [P_FDR_(M_He_)] mechanisms were directly estimated from the total interference model functions as P_SDR_(M_He_) = 2d and P_FDR_(M_He_) = (1 − d). The probabilities of a marker being inherited as homozygous under SDR and FDR were estimated as P_SDR_(M_Ho_) = (1 − 2d) and P_FDR_(M_Ho_) = d, respectively.

Therefore, the LOD values used to compare the probabilities of a heterozygous or a homozygous diploid gamete occurring at a locus, under the two models (SDR/FDR), were calculated respectively as:
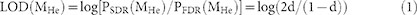
and
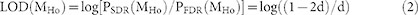


For each restitution model, the probability of a single unreduced gamete [P(G)] presenting the observed allelic configuration for *i* unlinked markers (M_i_) is the product of the probabilities of the observed genotype at each locus, P(G) = πP_Mi_, and therefore the LOD value to compare the SDR/FDR models is the sum of the LOD at each locus,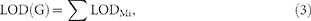 where P_Mi_ and LOD_Mi_ are the probability and the LOD value of the observed genotype at the locus *I*, respectively.

As an example, if three unlinked loci (M_1_, M_2_ and M_3_) were heterozygous, homozygous and homozygous, respectively, the probabilities of observing such gametes [P(G); (M_1He_–M_2Ho_–M_3Ho_)] are, respectively,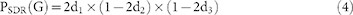 under SDR and
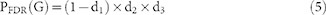
under FDR

The LOD value used to compare the probabilities of SDR/FDR models is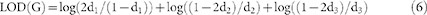 where *d_i_* is the distance from the locus *i* to its centromere.

LOD scores greater than 3 (the probability of the observed gamete is more than 1000-fold higher under the SDR model than the FDR one; LOD3) or greater than 2 (the probability of the observed gamete is more than 100-fold higher under the SDR model than the FDR one; LOD2) were considered as thresholds indicating that SDR was the mechanism involved in the single unreduced gamete formation, whereas LODs below −3 (or −2) indicate that FDR was the underlying mechanism; for LOD scores between −3 and 3 (or between 2 and −2), we considered that the mechanism could not be determined significantly.

#### Identification of the restitution mechanism at population level

Considering an infinite population of 2*n* gametes and a single locus, the probability of observing a sample of gametes [P(Pop)] with *j* heterozygous and *k* homozygous individuals under the SDR and FDR model are, respectively:
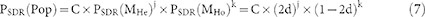

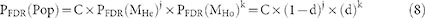
where C is a combinatory coefficient constant for the observed sample. Therefore,
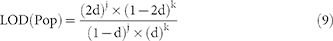


If *i* independent loci are analysed, the probabilities of the observed sample of gametes occurring under the SDR [P_SDR_(Pop)] or FDR [P_FDR_(Pop)] models are the products of the probabilities of the observed sample at each locus
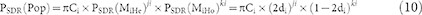

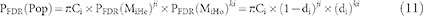
 and therefore,
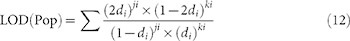
where P(M_iHe_),P(M_iHo_), *ji*, *ki*, _and_ d_i_ are, respectively, the probability of heterozygous individuals, probability of homozygous individuals, number of heterozygous individuals, number of homozygous individuals and distance to centromere for the locus *i*.

At the population level, LOD scores greater than 3 were considered to indicate that SDR was the mechanism involved in unreduced gamete formation, whereas LODs below −3 indicated that FDR was the underlying mechanism. When LOD scores between −3 and 3 were obtained, we considered that the mechanism could not be significantly determined.

#### Studies to check the power of the method

We assessed the power of our method using simulated samples of diploid gametes arising from either the FDR or SDR mechanisms. From a theoretical infinite population with heterozygous and homozygous genotype frequencies directly defined by the considered locus-centromere distances [(P_FDR_(M_He_) = (1 − d); P_FDR_(M_Ho_) = d; P_SDR_(M_He_) = 2d; P_SDR_(M_Ho_) = (1 − 2d)] as explained above), individual gametes with information for nine markers (the haploid number of chromosome in *Citrus*) were randomly generated. Then, the LOD values of these gametes were calculated as described above. We estimated the proportion of gametes with significant solutions at LOD3 (LOD value> 3 or <−3) and LOD2 (LOD value> 2 or <−2) when analysing 1–9 markers mapped at the same centromere distance, but in different chromosomes, and for distances ranging from 0 to 20 cM.

Gamete populations were also generated in order to estimate the theoretical number of hybrids that would need to be analysed to obtain significant conclusions for a mechanism, depending on the number of markers used and the marker-centromere distances. From each theoretical population (FDR and SDR populations), 200 replicates of populations (with 1–100 gametes/population) were randomly generated. The generated population LODs were calculated as described above and, for each number of considered markers at a given centromere distance, we identified the minimum number of gametes needed in order to be able to reach a true significant conclusion for at least 99% of the generated populations (99% of replicates with LOD> 3 for SDR or LOD <−3 for FDR).

From 1000 randomly selected gametes with nine independent markers (at the same distance from their respective centromere) from a theoretical SDR and FDR infinite population, we analysed the percentage of replicates with significant LOD value (i.e., LOD3 and LOD2) at a given distance considering the data from 1–9 markers.

**Figure 3:**
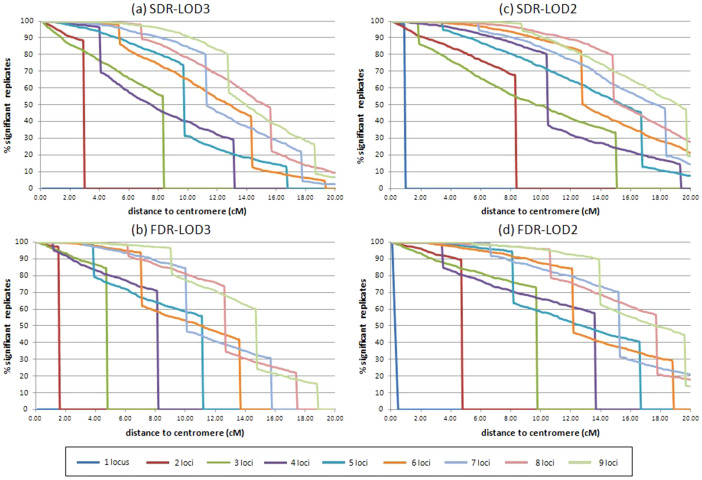
Percentage of replicates with significant LOD value considering a LOD3 for (a) theoretical SDR and (b) FDR populations, and considering a LOD2 for (c) SDR and (d) FDR populations.

Curves corresponding to a significant true answer are shown in Figure 3. All curves display a vertical drop to 0, corresponding to the distance when the maximum theoretical LOD score (when all considered markers are in the most favourable combination for the model) is below the considered threshold. Compared with LOD3, the LOD2 threshold allows maintenance of the progressive decrease of the significant answer with increasing distance. As distance increases, more markers are needed to maintain a high level of significance.

At LOD3, the usefulness of only one marker is null for both the SDR (Figure 3a) and the FDR (Figure 3b) models at a very low marker distance from the centromere (0.1 cM). At 5 cM, at least five (for SDR) and six (for FDR) markers are necessary to maintain a 90% true significant identification of the mechanism. When all markers were at least 10 cM from centromeres, nine markers were necessary to provide a 90% true significant answer for the SDR population, but only 78% significant true answers were obtained with nine markers for a FDR population. At 15 cM and nine markers, the true identification rates fall to 44% and 24% for SDR and FDR, and, at 20 cM, to 6.6% and 0%, respectively.

If the LOD2 threshold is considered, a single marker was informative in the first cM interval for the SDR model (Figure 3c) but significant replicate number decreases very quickly for FDR (Figure 3d). At 5 cM, at least four and five markers were necessary to provide 90% of true significant identification for SDR and FDR populations, respectively. With all markers at 10 cM from centromeres, at least eight markers were necessary to provide 90% true significant answers with an SDR or FDR population. For nine markers, the rate of true significant identification is improved for the SDR population at 15 cM and 20 cM (70% and 19%, respectively) as well as for the FDR population (59% and 14%, respectively) when compared with LOD3.

The rate of false identification (FDR significant conclusion [i.e., LOD <−3 or LOD <−2] for a SDR population, or reciprocally) is very low for both models (SDR or FDR), whatever the centromere distance and the number of considered loci. At LOD3, it is under 0.1% for all conditions and it remains below 1% for the LOD2 threshold (Figure S1).

**Figure 4:**
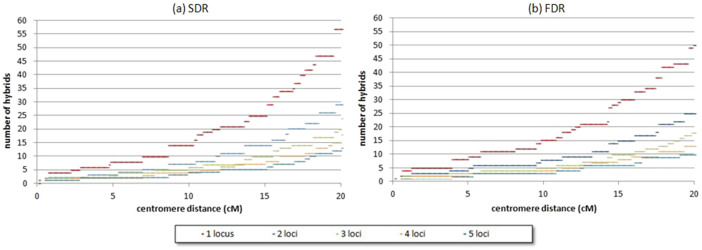
Number of hybrids needed to obtain significant conclusions for (a) SDR and (b) FDR mechanisms.

At the population level (Figure 4), due to the probabilities of the 2*n* gamete genotypic structure under FDR and SDR models becoming similar as the distance to centromere rises, the number of hybrids needed to obtain significant conclusions for a mechanism increases as an exponential function and is more pronounced when analysing a single marker only.

For a concrete locus-centromere distance, the number of hybrids (*h_m_*) needed is related to the number of markers analysed as: *h_m_* *=* *h_1_/m*, being *h_1_* the number of hybrids needed for one marker and *m*, the number of markers analysed. For example, for a SDR population model, at 20 cM, 58 hybrids are necessary if analysing only one marker, 29 are necessary for two markers, and 20 are necessary for three markers. The number of hybrids needed to provide the same level of conclusive answer is slightly lower for FDR (50 hybrids for one marker at 20 cM). With these population sizes, no false mechanism identification occurred for the generated populations.

### Inference of allelic configuration of triploid hybrids and corresponding 2*n* gametes

Assignment of allelic configuration in heterozygous triploid hybrids was performed using the MAC-PR method for SSR markers^55^ (Figure S2) adapted for *Citrus* by Cuenca *et* *al.*^37^. However, this method uses a 1:1 dosage correction from the relative allele signals for heterozygous diploid parents (A_1_:A_2,_ A_1_:A_3_ or A_3_:A_4_). Therefore, for markers displaying A_1_A_2_ × A_1_A_3_ configuration in the parents, among the heterozygous triploid hybrids only the A_1_A_2_A_2_/A_1_A_1_A_2_ or A_1_A_3_A_3_/A_1_A_1_A_3_ configurations can be determined using these methods, while no direct allele dosage estimation can be obtained for a triploid with A_2_/A_3_ heterozygosity without a reference for the relative A_2_/A_3_ allele signal. Similarly, for markers displaying the A_1_A_2_ × A_3_A_4_ configuration, it is not possible to directly estimate allele dosage for the heterozygous triploid hybrids. In these situations, it is possible to use a 1:1 dosage correction between A_1_ and A_3_ (for example) from the peak ratios of A_1_A_2_A_3_ triallelic hybrids observed in the same family.

A concrete example can be the genotype assignment for the “Ellendale × Fortune” population (Additional file 1) and the mCrCIR07F11 marker. “Ellendale” shows 160/162 alleles, and “Fortune” 152/164 alleles. Hybrid#1 shows 152/160/162 allele configuration. This situation allows a 1:1 dosage correction for relative allele signals between 152/160 and 152/162. Similarly, hybrid #11 shows 160/162/164 allele configuration for the same marker, and therefore, allows using a 1:1 dosage correction for relative allele signals between 160/164 and 162/164. All this 1:1 dosage corrections allow inferring the allele dosage for this marker in the remaining hybrids within this population.

### Identification of the unreduced gamete parental origin

For each hybrid, determination of the 2*n* gamete origin was carried out by identifying the parent that passed double genetic information to the hybrid. For markers displaying A_1_A_2_ × A_1_A_1_ or A_1_A_2_ × A_1_A_3_ configurations, the identification of A_1_A_2_A_2_ or A_2_A_2_A_3_ (i.e., double dosage of A_2_, the allele specific to the female parent) configurations in the hybrid would imply a female origin of the 2*n* gamete. For the second combination, the observation of A_1_A_3_A_3_ or A_2_A_3_A_3_ (i.e., double dosage of A_3_, the allele specific to the male parent) would indicate a male origin.

For markers displaying A_1_A_2_ × A_3_A_3_ configurations in the parents, the identification of A_1_A_2_A_3_, A_1_A_1_A_3_, or A_2_A_2_A_3_ configurations in the hybrid resulted from a maternal origin of the unreduced gamete, while A_1_A_3_A_3_ or A_2_A_3_A_3_ resulted from a paternal origin.

For markers with A_1_A_2_ × A_3_A_4_ parental configuration, the identification of the following genotypes (A_1_A_1_A_3,_ A_1_A_1_A_4,_ A_1_A_2_A_3,_ A_1_A_2_A_4,_ A_2_A_2_A_3,_ A_2_A_2_A_4_) and (A_1_A_3_A_3,_ A_2_A_3_A_3,_ A_1_A_3_A_4,_ A_2_A_3_A_4,_ A_1_A_4_A_4,_ A_2_A_4_A_4_) implied, respectively, female and male origin of the 2*n* gamete.

Once the parental origin of the 2*n* gamete was identified, the inference of the allelic configurations of the unreduced gametes from triploid hybrid genotyping was carried out as previously described by Cuenca *et* *al.*^37^^.^

A summary of triploid genotypes allowing inference of the 2*n* gamete genotype and origin, either directly or by inferring allele doses from diploid parents or reference triploid hybrids, is given in additional table S1. Loci with complete differentiation between the parents (A_1_A_2_ × A_3_A_4_ or A_1_A_2_ × A_3_A_3_) are by far the best configurations as they allow unequivocal identification of the 2*n* gamete parent and unambiguous determination of 2*n* gamete structure. When the parental origin of a 2*n* gamete has been determined by triploid patterns at other loci, the 2*n* gamete structure can be inferred for all triploid hybrids for the loci sharing a single allele between the two parents.

Following the previous example for the “Ellendale × Fortune” population (Additional file 1), hybrid #1 shows 152/160/162 allele configuration for the mCrCIR07F11 marker. This situation allows the unequivocal identification of the maternal parent as the 2*n* gamete producer for this hybrid. Similarly, the observed configurations for the rest of the hybrids within this population (152/**160**, 152/**162**, **160**/164 and **162**/164) allow the identification of the maternal parent as the 2*n* gamete producer for all hybrids with information for this marker. Once the female parent has been identified as the 2*n* gamete producer for a hybrid, for example hybrid#1, we can infer the 2*n* female gamete and male gamete configurations from the allelic and dosage observations for the other markers. In the situation that it is not possible to infer the 2n gamete producer (hybrids #30, #36, #57 and #69), additional markers have been analysed.

In this work, 543 citrus triploid hybrids were analysed and allelic patterns of the markers (Additional file 1) allowed unequivocal identification of the origin of the double dosage for each analysed triploid hybrid. Female parents were the unreduced gamete producers leading to triploid hybrids for all studied parental combinations. No triploid hybrid arising from unreduced pollen was found. It was therefore possible to infer the maternal 2*n* gamete genotypes for all hybrids and loci.

### Identification of the restitution mechanism at the individual level in citrus

Between 4 and 7 SSR and InDel markers have been used to analyse all 543 triploid hybrids. Allelic segregation for homozygous diploid gametes has been analysed within each family by a chi-squared test. Some markers deviated from the 1:1 expected ratio in populations with a reduced number of hybrids. Considering population with more than 20 hybrids, only the mCrCIR06B05 marker in “Fortune”-derived populations (χ^2^ = 5,531; p-value = 0,018) and for the CF-ACA01 and CI07C07 markers in “Hernandina × Nadorcott” population (χ^2^ = 9,524; p-value = 0,002 and χ^2^ = 6,737; p-value = 0,009, respectively) showed significant segregation distortions.

Heterozygosity restitution ranged between 0% and 100% for the analysed 2*n* gametes, with a mean value of 14,87%, whereas for markers, HR ranged between 0% and 54%, with a mean value of 15,49%. Distribution of HR for both hybrids and markers is clearly biased to values near 0% (Table S3).

**Figure 5:**
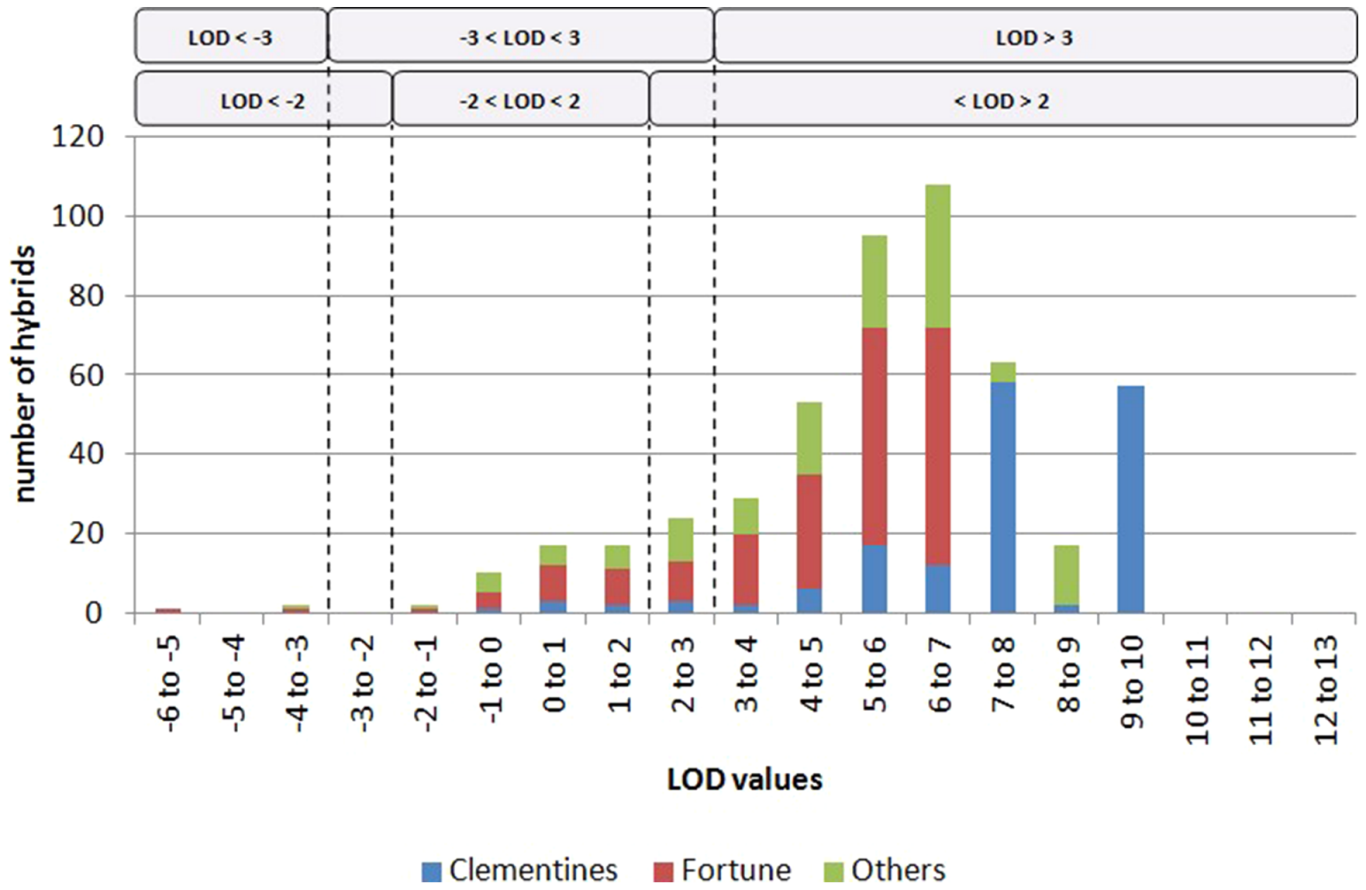
Frequency histogram of LOD values obtained for each individual 2*n* gamete, indicating those arising from clementines, ‘Fortune’ mandarin and other mandarins analysed in this study.

LOD score testing the SDR/FDR hypothesis was estimated for each individual 2*n* gamete from its inferred genotype, as described in the statistical method section. Positive LODs were found for 523 hybrids of the 543 analysed (Figure 5), suggesting a large global predominance of the SDR mechanism. The LOD distribution for clementine 2*n* gametes is displaced to higher values when compared with the distribution for ‘Fortune’ and other mandarin 2*n* gametes Fifty-seven diploid gametes occur with LOD between 9 and 10, and these correspond mostly to the ‘Fina’ clementine progeny (Figure 5).

When using LOD3 as the threshold, SDR was found to be the restitution mechanism underlying unreduced megagametophyte production for 424 (85.3%) of the analysed triploid hybrids (Table 1). For one triploid hybrid arising from ‘Ellendale’ and two arising from ‘Fortune’ (0.6%), the FDR mechanism was implicated. The other 70 (14.1%) triploid hybrids did not give significant conclusions for either the SDR or FDR mechanisms. All unreduced gametes arising from ‘Encore’, ‘Fallgo’, ‘Guillermina’, ‘Honey’, ‘Loretina’ and ‘Wilking’ were identified as having an SDR origin, whereas for 33 unreduced gametes arising from ‘Fortune’ (16.7%) no significant conclusions were obtained (Table 1).

When using LOD2 as the threshold, the percentage of gametes with unidentified origins decreased to 9%. Gametes attributed to SDR increased to 90.1%, with significance achieved for an additional three clementine gametes, another ten from ‘Fortune’ and an extra 11 from other mandarins. No additional 2*n* gametes arising from FDR were identified.

### Identification of the restitution mechanism at population level in citrus

At the population level, all LOD scores were greater than 3, even for small populations with fewer than five hybrids. Therefore, SDR was identified as the preeminent restitution mechanism producing 2*n* megagametophyte for all female parents analysed (Table 1).

## Discussion

### A powerful maximum-likelihood method to compare FDR and SDR hypothesis at the individual and population level has been developed

In sexual polyploidisation, polyploids are generated by the formation of unreduced diploid gametes. From the cytogenetic point of view, two types of meiotic nuclear restitution leading to 2*n* gamete formation are considered, FDR and SDR^5^,^9^,^56^,^57^.

The identification of the meiotic restitution mechanisms driving the formation of unreduced gametes is complex. However, molecular marker analysis is useful in such identification, and several methods, generally assuming complete chiasma interference, have been developed previously. The method proposed by Cuenca *et* *al.*^37^, based on the HR restitution curve along a linkage group, allows simultaneous identification of the restitution mechanism, raw centromere location, and comparison of several chromosome interference models. This approach is based on the analysis of genotype frequency in relatively large populations and provides global results of the preeminent mechanism; however, determination of the potential coexistence of the two mechanisms in the same progeny was not possible.

In this study, a maximum-likelihood approach based on marker HR with centromeric loci was developed and successfully applied both at the individual and population levels. Knowledge of marker-centromere distances greatly improves the statistical power of the comparison between the SDR and FDR hypotheses. For example, in this study, the restitution mechanism was identified in ‘Fortune’ as SDR at the population level with a LOD(SDR/FDR) of 933, whereas for the same population using 12 markers without information regarding marker-centromere distance, but with HR values under 50%^37^, the mechanism was identified as SDR with a LOD value of only 6.8. With the method proposed in the present paper, conclusions at the population level could therefore be obtained from smaller numbers of progeny and fewer markers than with non-located markers.

The theoretical limits of our method were assessed by the simulation of populations arising from FDR or SDR mechanisms. At the population level, considering that the independent markers used are at the same distance from their respective centromeres, the power of the statistical test was directly linked to the product of the number of markers and the number of individuals. That means that the efficiency would be the same for *n* individuals with *m* markers as for 2*n* individuals with *m*/2 markers. Moreover, the necessary *n·m* genotyping points increase exponentially with increasing distance of the marker to the centromere. For example, to obtain a significant answer higher than 99%, it would be necessary a *n*·*m* higher than fifty-seven for markers at 20 cM, while a *n·m* value higher than eight and four would be sufficient for markers at 5 cM and 1 cM, respectively. The selection of markers as close as possible to their centromere is therefore a key element for successful analysis when low numbers of individuals and markers are used.

In the study of citrus 2*n* gamete progenies, significant results were obtained for all analysed populations, even for populations lower than five individuals.

One major improvement of our approach over existing methods is that it allows the identification of the restitution mechanism for each individual unreduced gamete. Simulation studies indicated that the proximity of markers to the centromeres is a key factor. With markers closer than 5 cM, five markers are sufficient to result in 95% significant answers, but significance diminishes to less than 78% and 0% for nine markers at 10 cM and 20 cM from their centromeres, respectively.

The importance of selecting markers very close to the centromere to obtain significant conclusions at the individual level is illustrated by the results of our citrus analysis. Indeed, a very high percentage of significant results at the individual level (95.4%) and with high LODs were obtained for the ‘Fina’ clementine progeny analysed with markers closer to centromeres than the other progenies.

Other mechanisms than meiotic restitution, also leading to unreduced gamete formation have been described, like pre-meiotic and post-meiotic genome doubling. However, both these mechanisms have only rarely been documented in plants^4^. Nevertheless, genetic configurations of the resultant unreduced gametes would be different than FDR or SDR-gametes.

In animals, pre-meiotic genome doubling leads to parthenogenesis^58^. Doubled chromosome number is reduced through meiosis and the resulting daughter chromosomes pair in the first meiotic prophase with their genetically identical counterpart. As a result, the genotype of the parent is passed on to the offspring unchanged. Analysing centromeric markers, this situation could be confused with FDR mechanism, if all markers resulted fully heterozygous in the offspring. However this situation was observed for only one of the 543 citrus diploid gametes analysed in the present work.

In case of post-meiotic doubling, meiotically formed haploid spores undergo an extra round of genome duplication, and consequently yield fully homozygous 2*n* gametes. This situation could be also obtained in case of SDR, if all analysed centromeric markers resulted fully homozygous in the offspring. In the present work, 268 unreduced gametes resulted fully homozygous, but some heterozygous loci were observed in other unreduced gametes within the same populations, discarding a complete post restitution model at population level. At individual level, the analysis of telomeric markers allow analysing if homozygosity is maintained along the chromosome arm, and therefore concluding if the diploid gametes resulted from post-meiotic doubling or SDR. As an example, out of the 87 diploid gametes of “Fina” clementine analysed in the present study, 58 were totally homozygous for the 6 centromeric loci analysed. However, for the same population analysed with 104 markers including centromeric and telomeric loci, the HR at individual level ranged between 25% and 65%^36^. This broader marker study totally discard the pre- and post-meiotic doubling mechanisms at individual level. Similarly, additional marker information for the other families (data not shown) discarded the pre- and post-meiotic doubling hypothesis.

### 2*n* megagametophytes arising from SDR are the preeminent source of triploid occurrence in 2*x* × 2*x* hybrid populations using mandarin-like parents

Spontaneous occurrences of citrus triploid hybrids arising from the union of 2*n* megagametophytes with haploid pollen have been noted since the seventies^34^,^32^,^59^. However, the frequency of such events is generally low^32^,^60^ and extensive breeding programs based on this type of hybridisation require very effective methodologies for embryo rescue and ploidy evaluation of large progenies mandarins^32^. To date, very few cases of citrus triploid hybrid occurrence in 2*x* × 2*x* crosses from unreduced pollen have been reported^35^,^38^; our unpublished results].

In this study, the mechanism leading to triploid formation in 2*x* × 2*x* crosses was elucidated, both at individual and population level, for nineteen varieties used as female parents.

All the 543 triploid hybrids analysed originated from 2*n* megagametophytes and, therefore, no 2*n* pollen contributed to the production of triploids in our parental combinations. These results expand to a large range of genotypes the prior conclusion obtained from cytological studies^34^,^57^ for ‘Sukega’ (*C. paradisi* × *C. sinensis*), ‘Temple’ (*C. reticulata* × *C. sinensis*) and clementine (*C. clementina*), indicating that in such 2*x* × 2*x* crosses, triploid embryos were associated with pentaploid endosperm. However, the occurrence of triploids arising from 2*n* pollen at very low rates has been previously reported in studies using molecular markers for three selections of clementine (‘Caffin’, ‘Commun’ or ‘SRA85’ and ‘Muskat’), ‘King’ mandarin pollinated with *C. deliciosa* (‘Tardivo di Ciaculi’, ‘Willow Leaf’), *C. reticulata* (‘Hansen’, ‘Ananas’), *C. paradisi* (‘Star Ruby’) and *C. sinensis* (‘Tarroco Rosso’, ‘Sanguinelli’)^35^ and for *C. sinensis* × *Poncirus trifoliata* hybridisations^38^.

When using the LOD3 threshold, SDR was identified as the restitution mechanism for 85.3% of the analysed triploid hybrids, no significant conclusions were obtained for 14.1% of the hybrids, and 0.6% of the analysed triploids were derived from FDR (one triploid hybrid arising from ‘Ellendale’ and two arising from ‘Fortune’). When the LOD2 threshold was considered, the percentage of individuals with unidentified origin decreased to 9% and SDR levels increased to 90.1%. Moreover, we conducted individual level analysis of previously studied ‘Fortune’ mandarin progeny^37^ and the progeny arising from ‘Fina’^36^, and we confirmed SDR at the individual level for most hybrids, which concurs with the global-level conclusions proposed in these two studies. In the current study, six clementine genotypes were also analysed to discover their unreduced gamete formation mechanism. Results indicate that SDR is the most probable mechanism in the clementine group, in agreement with previous conclusions of Luro *et* *al.*^35^. For the other mandarin varieties, SDR was also the most probable mechanism at the individual level and, therefore, also at the population level. Taken together, our data and those of others suggest that SDR is the major mechanism underlying unreduced megagametophyte formation in most mandarin genotypes.

The mechanism leading to unreduced eggs or pollen was previously elucidated for several plant species^4^,^12^. Bretagnolle and Thompson^5^ identified that both FDR and SDR are responsible for 2*n* pollen formation, while SDR is more frequent in the formation of 2*n* eggs. In potato, 2*n* pollen arises predominantly by FDR^16^, while 2*n* megagametophytes arise most frequently by SDR^61^, although SDR-FDR mixture in the formation of 2*n* eggs has been also found^62^. Bilateral sexual polyploidisation can arise either from FDR and SDR in *Lilium*^8^,^47^,^63^ and alfalfa^22^. Moreover, other examples of plant species where FDR and SDR may occur simultaneously has been described^5^, underlining the influence of genotype and environment on the expression of meiotic abnormality factors^64^,^65^.

### Implications for citrus triploid breeding

The genetic and phenotypic consequences of FDR and SDR gametes are highly divergent, and are of potential importance for breeding applications, due to the different parental heterozygosity rate that each mechanism transmits to the polyploid progeny^4^.

Under FDR, the resulting 2*n* gametes are heterozygous from the centromere to the first crossover point, and hence the gametes retain most parental heterozygosity and epistatic interactions. With the SDR mechanism, the resulting 2n gametes are homozygous from the centromere to the first crossover point, but retain parental heterozygosity on the telomeric regions^12^. As a result, SDR-2*n* gametes confer a lower level of heterozygosity than FDR-2*n* and show a corresponding greater loss of parental epistasis^5^,^66^.

If an SDR origin of 2*n* gametes is assumed for most mandarins, sexual polyploidisation may lead to a reduced average of HR and, therefore, loss of epistatic interactions. Therefore, when compared with interploid crosses using doubled diploids^67^,^68^, the sexual polyploidisation strategy should produce more polymorphic progeny by creating a larger number of new multilocus allelic combinations^4^. This provides the opportunity to select innovative products within the perspective of market segmentation as a commercial strategy.

Consequences of the SDR restitution mechanism would be clearly apparent for a character controlled by a single gene. If the gene is heterozygous in the female parent, most unreduced gametes will be homozygous for that gene if it is located near the centromere, but gametes will be mostly be heterozygous for the gene if it is telomere-proximal (partial interference model;^37^). Recently, Cuenca *et* *al*.^69^ analysed the inheritance of resistance to *Alternaria* brown-spot fungal disease in citrus triploid progenies arising from crosses between diploid parents. They demonstrated that the resistance was controlled as a recessive trait by a single locus located near a centromere (10.5 cM from the centromere of chromosome 3). If a susceptible female parent is heterozygous, the SDR mechanism leads to approximately 80% homozygous unreduced gametes, half of having two resistant alleles. As *Alternaria* resistance is a major selective trait when maternal heterozygous parents are used, sexual polyploidisation is a more effective strategy than the use of interploid crosses, which will result in only 16.7–22.5% of progeny being resistant.

For dominant traits controlled by a single centromeric locus, interploid crosses should be more interesting than 2*x* × 2*x* crosses. For characters controlled by major loci more distant than 30 cM from the centromere, the efficiency of the two triploid breeding strategies would be relatively similar. This information is now being used routinely in the mandarin triploid breeding program carried out in Spain^70^.

## Methods

### Plant materials

Analyses were performed using 543 triploid hybrids derived from 19 different mandarin genotypes as female parents in 2*x* × 2*x* cross populations (Table 2). The mandarin genotypes include six clementine and 13 hybrid mandarins. Triploid hybrids were grown at the ‘Instituto Valenciano de Investigaciones Agrarias’ orchards in Moncada, Valencia, Spain. Practical details for the establishment of triploid populations from 2*x* × 2*x* crosses by embryo rescue and triploid selection by flow cytometry can be found in Aleza *et* *al.*^32^. All triploid genotypes in the present study were selected after ascertaining their hybrid nature by molecular marker analysis (data not shown). Taxonomic information about both female and male parental accessions is given in additional Table S2 according to the standard classification system for the *Citrus* genus^71^,^72^.

### Selection of centromeric markers for the analysis of 2*n* gamete origin and formation mechanisms

Triploid citrus hybrids obtained in 2*x* × 2*x* hybridisations arise from unreduced megagametophytes^32^,^33^,^34^,^35^,^59^,^60^. Therefore, markers heterozygous for the female parent and displaying polymorphism between the two parents were primarily selected for the molecular characterisation of triploid hybrids and analysis of 2*n* gamete origin.

Centromere positions in all nine clementine chromosomes are known^36^. Molecular markers within 20 cM of the centromere were used in this study because centromere-proximal markers are more informative with regard to the mechanisms of 2*n* gamete formation than centromere-distal markers^53^. Within this range, the lowest expected HR rate is greater than 80% for FDR, while the highest HR for SDR is 40% (Figure 2). Twenty-five markers were selected for genotyping the triploid progeny. Between four and seven of these centromeric markers were used for genotyping each population (Table 3).

### Genotyping of triploid hybrids

#### DNA extraction

Leaf DNA of triploid hybrids and their parents was isolated using the Plant DNAeasy kit from Qiagen Inc. (Valencia, CA, USA), following the manufacturer’s protocol.

#### SSR and InDel analyses

Polymerase chain reactions (PCRs) were performed with wellRED oligonucleotides (Sigma-Aldrich®, St Louis, MO, USA) in a Mastercycler epgradient S (Eppendorf Scientific Inc., Westbury, NY, USA). The reaction (volume, 15 µl) contained 0.8 U Taq polymerase (Fermentas®, Burlington, VT, USA), 0.1 mM of each dNTP, 5 mM MgCl_2_, 3 mM of each primer, and 30 ng of DNA in buffer containing 750 mM Tris-HCl (pH 9), 50 mM KCl, 200 mM (NH_4_)_2_SO_4_, and 0.001% bovine serum albumin. The PCR program was 94°C for 5 min; 40 cycles of 30 s at 94°C, 1 min at 55°C and 30 s at 72°C, and a final elongation of 10 min at 72°C. Separation was carried out by capillary gel electrophoresis (CEQ 8000 Genetic Analysis System; Beckman Coulter Inc., Fullerton, CA, USA). Data collection and analysis were carried out with GenomeLab GeXP (Beckman Coulter Inc.) version 10.0 software. Identification of allele doses in heterozygous triploid hybrids was carried out using the MAC-PR method^55^ adapted for *Citrus* by Cuenca *et* *al.*^37^.

## Author Contributions

J.C. and P.A.: Contributed to plant recovery, analised the data and wrote the manuscript; J.J.: Contributed to plant recovery; A.G.L.: Contributed to the data analysis; Y.F.: Contributed to plant recovery; L.N. and P.O.: managed the work and contributed to write and revise the manuscript; J.C. and P.O.: implemented the statistical method.

## Supplementary Material

Supplementary Information Supplementary Information

Supplementary Information Supplementary Dataset 1

## Figures and Tables

**Figure 1:**
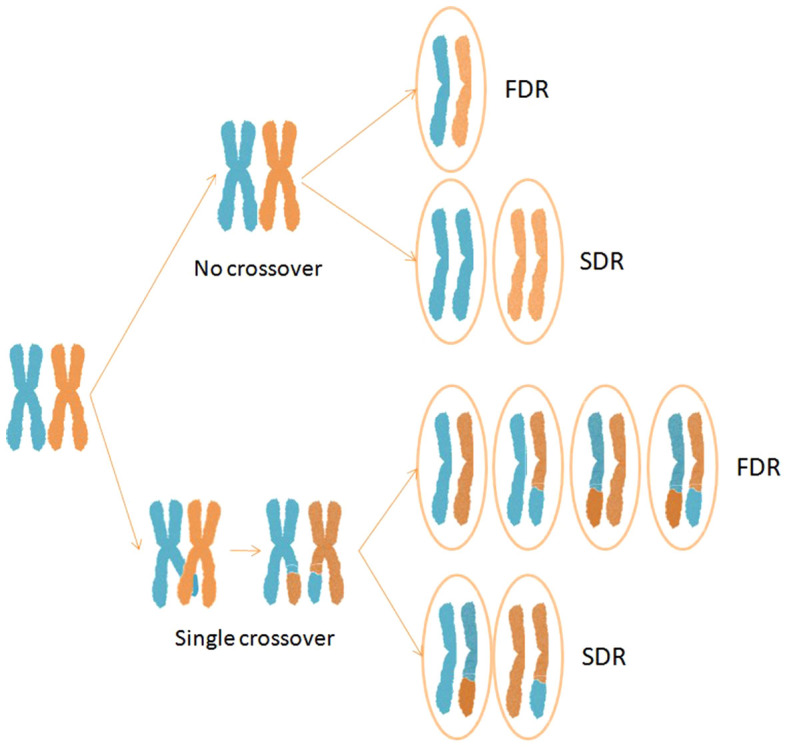
Half tetrads resulting from no crossover and single crossover events under FDR and SDR mechanisms of unreduced gamete formation.

**Table 1:** LOD scores for progeny of 19 female parents analysed at population level and individuals within each population originated by SDR, FDR, or with unidentified origin

Group	Female parent	Nh	Nm	Cd average (cM)	LODs> +3		LODs −3 − +3		LODs <−3		Population LOD P_SDR_/P_FDR_
					number	(%)	number	(%)	number	(%)	
Clementine	‘Bruno’	17	6	7.8	15	(88.2)	2	(11.8)	0	(0.0)	98.9
	‘Clemenules’	23	5	4.4	22	(95.6)	1	(4.4)	0	(0.0)	143.3
	‘Fina’	87	6	3.7	83	(95.4)	4	(4.6)	0	(0.0)	699.3
	‘Guillermina’	14	6	8.1	14	(100.0)	0	(0.0)	0	(0.0)	91.1
	‘Hernandina’	22	5	4.4	20	(90.9)	2	(9.1)	0	(0.0)	139.0
	‘Loretina’	2	7	9.5	2	(100.0)	0	(0.0)	0	(0.0)	10.3
Mandarin	‘Imperial’	24	5	5.0	23	(95.8)	1	(4.2)	0	(0.0)	138.5
Hybrid mandarin	‘Ellendale’	69	5	9.1	50	(72.5)	18	(26.1)	1	(1.4)	282.7
	‘Encore’	3	5	4.9	3	(100.0)	0	(0.0)	0	(0.0)	17.9
	‘Fallglo’	3	5	3.7	3	(100.0)	0	(0.0)	0	(0.0)	21.6
	‘Fortune’	197	5	6.8	162	(82.2)	33	(16.7)	2	(1.1)	933.0
	*‘Fortune’* *×* *‘Ellendale’*	*58*	*5*	*6.8*	*54*	*(93.1)*	*4*	*(6.9)*	*0*	*(0.0)*	*310.5*
	*‘Fortune’* *×* *‘Minneola’*	*35*	*4*	*5.2*	*28*	*(80.0)*	*6*	*(17.1)*	*1*	*(2.9)*	*145.7*
	*‘Fortune’* *×* *‘Murcott’*	*67*	*5*	*6.8*	*53*	*(79.1)*	*14*	*(20.9)*	*0*	*(0.0)*	*326.2*
	*‘Fortune’* *×* *‘Willowleaf’*	*37*	*4*	*6.6*	*28*	*(75.7)*	*8*	*(21.6)*	*1*	*(2.7)*	*150.6*
	‘Honey’	1	4	6.1	1	(100.0)	0	(0.0)	0	(0.0)	5.1
	‘Kiyomi’	21	5	6.3	20	(95.2)	1	(4.8)	0	(0.0)	162.9
	‘Moncada’	8	4	10.3	4	(50.0)	4	(50.0)	0	(0.0)	22.1
	‘Nadorcott’	11	4	8.9	6	(54.5)	5	(45.5)	0	(0.0)	23.9
	‘Orri’	29	5	10.2	17	(58.6)	12	(41.4)	0	(0.0)	84.5
	*‘Orri’* *×* *‘Fortune’*	*17*	*5*	*10.2*	*14*	*(82.3)*	*3*	*(17.7)*	*0*	*(0.0)*	*67.5*
	*‘Orri’* *×* *‘Oronules’*	*12*	*5*	*10.2*	*3*	*(25.0)*	*9*	*(75.0)*	*0*	*(0.0)*	*17.0*
	‘Ortanique’	6	5	6.6	6	(100.0)	0	(0.0)	0	(0.0)	28.1
	‘Umatilla’	5	4	11.3	1	(20.0)	4	(80.0)	0	(0.0)	9.6
	‘Wilking’	1	5	8.3	1	(100.0)	0	(0.0)	0	(0.0)	15.6

Nh: number of hybrids within each population (pop). Nm: number of markers analyzed over each population. Cd: Centromere distance. Italic format indicates different populations derived from ‘Fortune’ and ‘Orri’ female parents

**Table 2:** Number of hybrids within each population analysed in this study

# population	Population	Number of hybrids		# population	Population	Number of hybrids
1	Bruno × Chandler	17		10	Honey × N’15	1
2	Clemenules × Nadorcott	23		11	Imperial × Moncada	24
3	Ellendale × Fortune	69		12	Kiyomi × Nadorcott	21
4	Encore × Ellendale	3		13	Loretina × Chandler	2
5	Fallglo × N’15	3		14	Moncada × Ellendale	8
6	Fina × Nadorcott	87		15	Nadorcott × Ellendale	11
7	Fortune × 4 male parents	197		16	Orri × 2 male parents	29
*7a*	*Fortune* × *Ellendale*	*58*		*16a*	*Orri* × *Fortune*	*17*
*7b*	*Fortune* × *Minneola*	*35*		*16b*	*Orri* × *Oronules*	*12*
*7c*	*Fortune* × *Murcott*	*67*		17	Ortanique × Wilking	6
*7d*	*Fortune* × *Willowleaf*	*37*		18	Umatilla × Simeto	5
8	Guillermina × Chandler	14		19	Wilking × Fina	1
9	Hernandina × Nadorcott	22				

**Table 3:** Centromeric markers used for genotyping each triploid population

LG	Centromere Position (cM)	Marker id	Marker type	Reference	Marker Position (cM)	Centomere-distance (cM)	Populations analyzed (#)
1	60.66	mCrCIR06B05	SSR	73	50.27	10.39	7
		CID0806	InDel	74	55.17	5.49	8
		CIBE5720	SSR	75	58.45	2.21	4,5,11,12,16,17
		MEST539	SSR	In preparation	61.82	1.16	6
		MEST001	SSR	38	70.60	9.94	10,18,19
		mCrCIR07D05	SSR	37	75.60	14.94	1,13,14,17,18
2	56.87	CX2004	SSR	38	46.67	10.20	18
		CX6F23	SSR	38	49.53	7.34	1,2,4,5,6,7,9,10,11,12,13,15,16,17,19
3	90.59	CIBE4225	SSR	75	86.33	4.26	4,12
		CID5376	InDel	74	88.24	2.35	17
		MEST470	SSR	In preparation	88.76	1.83	6
		CX0124	SSR	In preparation	110.28	19.69	13,14,16
4	16.14	mCrCIR07D06	SSR	37	16.33	0.19	1,7,8,13
		CF-ACA01	SSR	In preparation	24.41	8.27	2,4,6,9,11
5	23.12	CID0245	InDel	In preparation	20.94	2.18	2,5,6,9
		MEST104	SSR	76	40.46	17.34	1,3,8,12,13,14,15,16,18,19
6	6.4	MEST191	SSR	In preparation	10.86	4.46	1,5,8,10,11,12,13,15,16,19
7	96.43	mCrCIR03B07	SSR	37	83.39	13.04	7
		CX0114	SSR	In preparation	94.97	1.46	3
		CI07C07	SSR	73	98.02	1.59	2,3,6,9,10
8	54.21	mCrCIR07B05	SSR	73	31.70	22.51	3
9	52.16	mCrCIR07F11	SSR	77	49.57	2.59	1,2,3,4,5,8,9,10,11,13,14,15,17,18,19
		CI08C05	SSR	73	55.14	2.98	7
