# Supplementary material for: Maximum-likelihood method identifies meiotic restitution mechanism from heterozygosity transmission of centromeric loci: application in citrus
Source: Sci Rep. 2015 Apr 20;5:9897. doi: 10.1038/srep09897 (PMC4403285; doi:10.1038/srep09897)

**Suplementary information**

**Title:**

**Maximum-likelihood method identifies meiotic restitution mechanism from heterozygosity transmission of centromeric loci: application in citrus**

**Authors:**

**José Cuenca, Pablo Aleza, José Juárez, Andrés García-Lor, Yann Froelicher, Luis Navarro, Patrick Ollitrault.**

**Table S1**. Inferring of the 2*n* gamete parental producer depending on parental configuration and the raw genotype pattern observed.

| **Parental configuration** | **Raw genotype pattern observed** | **Allele doses estimation method** | **Inferred triploid genotype** | **Inferred 2*n* gamete configuration** | **Inferred parental origin** |  | **Inferred 2*n* gamete if maternal origin proved** | **Inferred 2*n* gamete if paternal origin proved** |
| --- | --- | --- | --- | --- | --- | --- | --- | --- |
| **A_1_A_2_ x A_3_A_4_** | A_1_A_2_A_3_ | D | A_1_A_2_A_3_ | A_1_A_2_ | M |  | A_1_A_2_ |  |
|  | A_1_A_2_A_4_ | D | A_1_A_2_A_4_ | A_1_A_2_ | M |  | A_1_A_2_ |  |
|  | A_1_A_3_A_4_ | D | A_1_A_3_A_4_ | A_3_A_4_ | P |  |  | A_3_A_4_ |
|  | A_2_A_3_A_4_ | D | A_2_A_3_A_4_ | A_3_A_4_ | P |  |  | A_3_A_4_ |
|  | A_1_A_3_ | TH | A_1_A_1_A_3_ | A_1_A_1_ | M |  | A_1_A_1_ |  |
|  |  | TH | A_3_A_3_A_1_ | A_3_A_3_ | P |  |  | A_3_A_3_ |
|  | A_1_A_4_ | TH | A_1_A_1_A_4_ | A_1_A_1_ | M |  | A_1_A_1_ |  |
|  |  | TH | A_4_A_4_A_1_ | A_4_A_4_ | P |  |  | A_4_A_4_ |
|  | A_2_A_3_ | TH | A_2_A_2_A_3_ | A_2_A_2_ | M |  | A_2_A_2_ |  |
|  |  | TH | A_3_A_3_A_2_ | A_3_A_3_ | P |  |  | A_3_A_3_ |
|  | A_2_A_4_ | TH | A_2_A_2_A_4_ | A_2_A_2_ | M |  | A_2_A_2_ |  |
|  |  | TH | A_4_A_4_A_2_ | A_4_A_4_ | P |  |  | A_4_A_4_ |
| **A_1_A_2_ x A_1_A_1_** | A_1_ | D | A_1_A_1_A_1_ | A_1_A_1_ | NI |  | A_1_A_1_ | A_1_A_1_ |
|  | A_1_A_2_ | DP | A_1_A_2_A_2_ | A_2_A_2_ | M |  | A_2_A_2_ |  |
|  |  | DP | A_1_A_1_A_2_ | NI | NI |  | **A_1_A_2_** | **A_1_A_1_** |
| **A_1_A_2_ x A_3_A_3_** | A_1_A_2_A_3_ | D | A_1_A_2_A_3_ | A_1_A_2_ | M |  | A_1_A_2_ |  |
|  | A_1_A_3_ | TH | A_1_A_1_A_3_ | A_1_A_1_ | M |  | A_1_A_1_ |  |
|  |  | TH | A_3_A_3_A_1_ | A_3_A_3_ | P |  |  | A_3_A_3_ |
|  | A_2_A_3_ | TH | A_2_A_2_A_3_ | A_2_A_2_ | M |  | A_2_A_2_ |  |
|  |  | TH | A_3_A_3_A_2_ | A_3_A_3_ | P |  |  | A_3_A_3_ |
| **A_1_A_2_ x A_1_A_3_** | A_1_A_2_A_3_ | D | A_1_A_2_A_3_ | A_1_A_2_ | M |  | A_1_A_2_ |  |
|  | A_1_ | D | A_1_A_1_A_1_ | A_1_A_1_ | NI |  | A_1_A_1_ | A_1_A_1_ |
|  | A_1_A_2_ | DP | A_1_A_1_A_2_ | NI | NI |  | **A_1_A_2_** | **A_1_A_1_** |
|  |  | DP | A_1_A_2_A_2_ | A_2_A_2_ | M |  | A_2_A_2_ |  |
|  | A_1_A_3_ | DP | A_1_A_1_A_3_ | NI | NI |  | **A_1_A_1_** | **A_1_A_3_** |
|  |  | DP | A_1_A_3_A_3_ | A_3_A_3_ | P |  |  | A_3_A_3_ |
|  | A_2_A_3_ | TH | A_2_A_2_A_3_ | A_2_A_2_ | M |  | A_2_A_2_ |  |
|  |  | TH | A_2_A_3_A_3_ | A_3_A_3_ | P |  |  | A_3_A_3_ |

D: direct visualisation of allelic constitution; DP: inference of allelic doses based on diploid parent reference; TH: inference of allelic doses based on at least one reference triploid hybrid; M: maternal; P: paternal; NI: not identified. Bold letters indicate gamete structures inferred when parental origin has been proved from other markers.

**Table S2.** Taxonomic information on parental genotypes used in this study

| **Group** | **Genotype** | **Swingle and Reece, 1967** | **Tanaka, 1977** |
| --- | --- | --- | --- |
| clementine | ‘Bruno’  ‘Clemenules’  ‘Fina’  ‘Guillermina’  ‘Hernandina’  ‘Loretina’ | *Citrus reticulata* | *Citrus clementina* |
| mandarin | ‘Imperial’ |  | *C. reticulata* |
|  | ‘Willowleaf’ |  | *C. deliciosa* |
| hybrid mandarin | ‘Ellendale’ | *C. reticulata* X *C. sinensis* | *C. reticulata* X *C. sinensis* |
|  | ‘Encore’ | *C. reticulata* | *C. nobilis* X *C. deliciosa* |
|  | ‘Fallglo’ |  | [*C. clementina* X (*C. paradisi* X *C. tangerina*)] X *C. temple* |
|  | ‘Fortune’ |  | *C. clementina* X *C. tangerina* |
|  | ‘Honey’ |  | *C. nobilis* X *C. deliciosa* |
|  | ‘Kiyomi’ | *C. reticulata* X *C. sinensis* | *C. unshiu* X *C. sinensis* |
|  | ‘Minneola’ | *C. reticulata* | *C. paradisi* X *C. tangerina* |
|  | ‘Moncada’ |  | *C. clementina* X (*C. unshiu* X  *C. nobilis*) |
|  | ‘Murcott’ | *C. reticulata* X *C. sinensis* | (*C. reticulata* X *C. sinensis*) |
|  | ‘N’15’ | *C. reticulata* | *C. clementina* X [(*C. reticulata* X *C. unshiu*)] |
|  | ‘Nadorcott’ | - | (*C. reticulata* X *C. sinensis*) X (unknown) |
|  | ‘Umatilla’ | *C. reticulata* X *C. sinensis* | *C. unshiu* X *C. sinensis* |
|  | ‘Simeto’ | *C. reticulata* | *C. unshiu* X *C. deliciosa* |
|  | ‘Wilking’ |  | *C. deliciosa* X *C. nobilis* |
| pummelo | ‘Chandler’ | *C. grandis* | *C. maxima* |

**Figure S1**. Percentage of significant replicates giving false answer considering a LOD3 for populations arising from (a) SDR and (b) FDR, and considering LOD2 for populations arising from (c) SDR and (d) FDR.


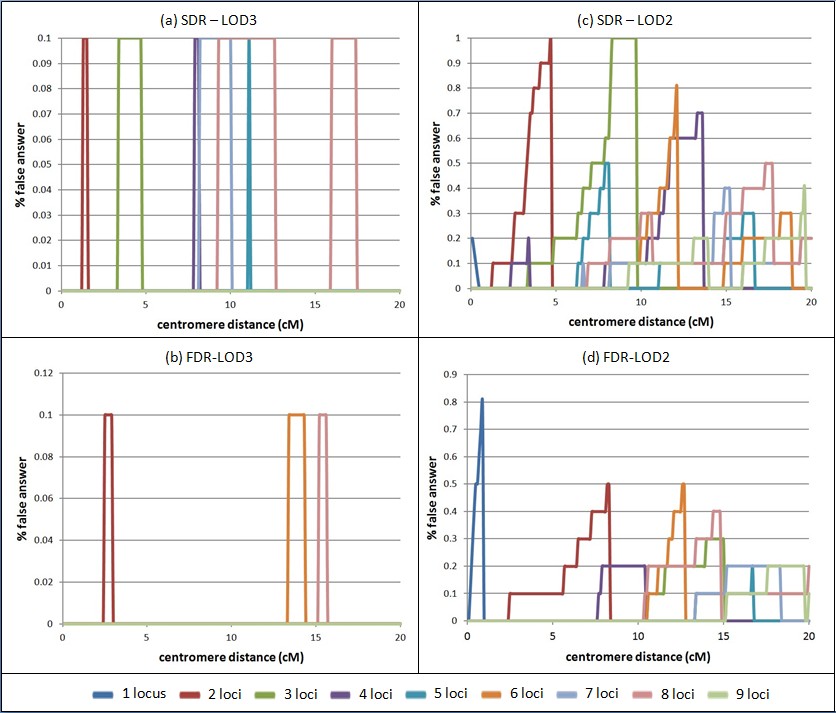


**Figura S2**. Assignment of allelic configuration in heterozygous triploid hybrids for SSR markers showing (A) A_1_A_2_ x A_1_A_1_ (B) A_1_A_2_ x A_3_A_3_ and (C) A_1_A_2_ x A_1_A_3_ parental configurations.


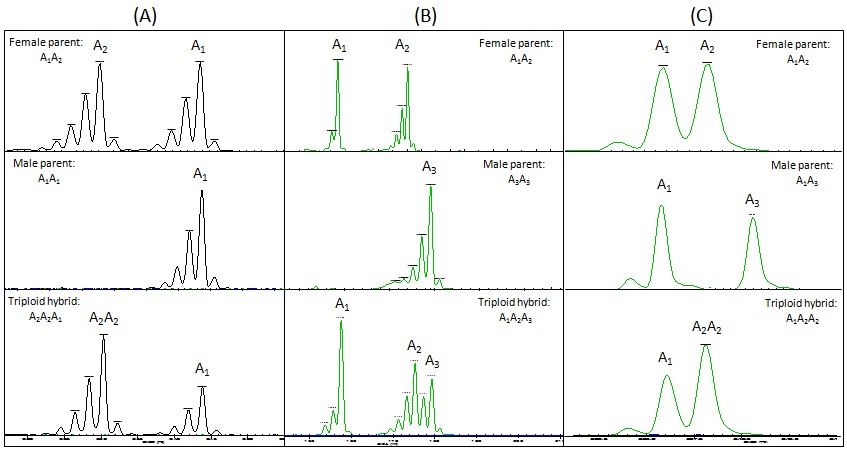


.

**Figura S3**. Distribution of the heterozygosity restitution for the hybrids and markers analysed.


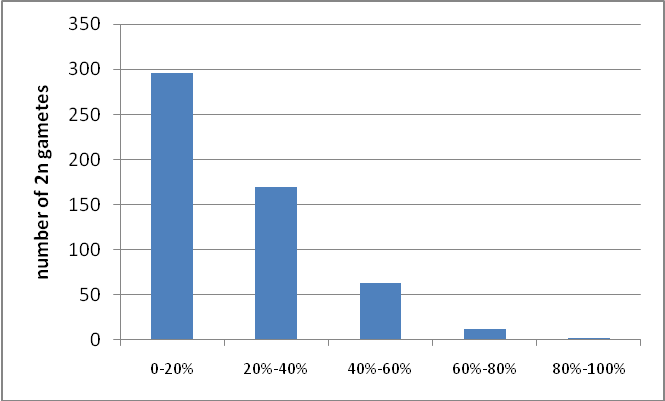

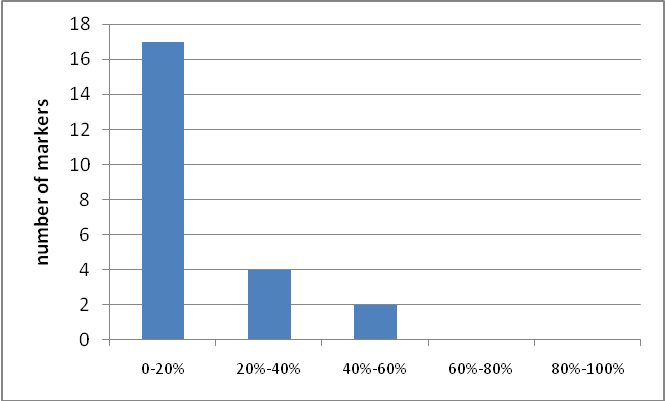

Supplement: Supplementary Information [file srep09897-s1.doc]
